# Supplementary material for: Pre-existing systemic and nasal antibodies against avian H5 influenza A viruses vary according to childhood imprinting
Source: mBio. 2026 Aug 27;17(10):e01892-26. doi: 10.1128/mbio.01892-26 (PMC13712818; doi:10.1128/mbio.01892-26)
Supplement: Supplementary file 1 [file mbio.01892-26-s0001.docx]

**Supplementary figures**

**Figure S1.** Sub-analysis of ELISA data excluding individuals whose samples were collected during the influenza vaccine season.

**Figure S2.** Gating strategy for the identification of H5 specific memory B cells. A/Fujian-Sanyuan/21099/2017 H5-specific B cells are shown after gating on IgG^+^IgD^-^CD19^+^CD3^-^CD8^-^CD16^-^CD14^-^CD10^-^ live single lymphocytes.

**Figure S3. Multiplex bead assay set up.** **(A)** IgG and IgA titration curves for H1, H2, H3, H1 stem, H5, the IAV NP coupled beads with serum and nasal fluid. Representative samples from 3 donors were tested for NP and H5, and from 1 donor for seasonal HAs. Dotted lines depict the working dilutions chosen for each sample type and antibody isotype respectively. **(B)** Total levels of IgG and IgA detected in each sample. Medians and 95% CIs (n=13/group) are shown, and p-values were determined by a Kruskal-Wallis test with Dunn's multiple comparisons test and were all >0.05.

**Figure S4. Serum and nasal fluid IgG and IgA against H5 and control antigens.** **(A)** IgG and IgA antibodies specific for H1, H2, H3, H1 stem, H5, the IAV NP and tetanus toxoid were determined in serum using a multiplex bead assay at sample dilution indicated in Figure S3A. Medians and 95% CIs (n=13/group) are shown, and p-values were determined by a Kruskal-Wallis test with Dunn's multiple comparisons test and only comparisons with p-values <0.05 are shown.  **(B)** Correlation between antibody titers to H5 or NP determined by ELISA (Figure 2) or by the bead multiplex assay (n=39). **(C)** IgG and IgA antibodies specific for H5 (A/Fujian-Sanyuan/21099/2017), the IAV NP and tetanus toxoid were determined in nasal fluid using a multiplex bead assay. Medians and 95%CI (n=13/group) are shown, and p-values were determined by a Kruskal-Wallis test with Dunn's multiple comparisons test and were all >0.05.
